# Training neural networks with universal adiabatic quantum computing

**DOI:** 10.3389/frai.2024.1368569

**Published:** 2024-06-21

**Authors:** Steve Abel, Juan Carlos Criado, Michael Spannowsky

**Affiliations:** ^1^Institute for Particle Physics Phenomenology, Durham University, Durham, United Kingdom; ^2^Theoretical Physics Department, CERN, Geneva, Switzerland; ^3^Departamento de Física Teórica y del Cosmos, Universidad de Granada, Granada, Spain

**Keywords:** adiabatic quantum computing, quantum computing, neural networks, binary neural networks, NN training

## Abstract

The training of neural networks (NNs) is a computationally intensive task requiring significant time and resources. This article presents a novel approach to NN training using adiabatic quantum computing (AQC), a paradigm that leverages the principles of adiabatic evolution to solve optimization problems. We propose a universal AQC method that can be implemented on gate quantum computers, allowing for a broad range of Hamiltonians and thus enabling the training of expressive neural networks. We apply this approach to various neural networks with continuous, discrete, and binary weights. The study results indicate that AQC can very efficiently evaluate the global minimum of the loss function, offering a promising alternative to classical training methods.

## 1 Introduction

Adiabatic quantum computing (AQC) is a paradigm of quantum computation that harnesses the principle of adiabatic evolution to solve computational problems (Farhi et al., 2001, 2002, 2006). In this approach, one must design a quantum Hamiltonian, whose ground state encodes the solution to the problem at hand. A quantum system with a time-dependent Hamiltonian is then employed to obtain this ground state. Initially, the Hamiltonian of this system should be simple enough for its ground state to be analytically known. Finally, it should coincide with the designed “target” Hamiltonian. The adiabatic theorem then guarantees that, if the evolution is sufficiently slow and the system is initialized in the ground state, it will remain in the ground state throughout the process. The computational prowess of AQC is equivalent to that of the conventional quantum computation model, implying that both models are polynomially equivalent (Aharonov et al., 2007). In other words, it is considered to be a universal quantum computing paradigm. Moreover, AQC has been realized experimentally in various systems, including solid-state single-spin systems under ambient conditions (Biamonte and Love, 2008; Xu et al., 2017). The purpose of this article is to demonstrate how AQC can be used to greatly enhance the training of neural networks (NNs).

Generally, NNs, similar to all self-adaptive optimization algorithms, consist of three core parts:

1. A system that encodes a complex function: typically, for an NN, this function is constructed as the composition of linear transformations and non-linear activation functions. The elements of the matrices that define these linear transformations are free parameters. When sufficiently many free parameters are present, they can be tuned so that the NN approximates any given function.
2. An output layer's loss function that dictates the NN's task: in the supervised learning framework, which we adopt in this article, one has a training dataset that provides a number of examples of desired inputs and corresponding outputs of the NN. The loss must then then be a function that is minimal when the outputs of the NN for the given inputs coincide with the desired outputs.
3. A training method to minimize the loss function: this may be any algorithm that tunes the free parameters of the NN so that they minimize the loss. In classical computing, this is usually performed through gradient descent-based methods.

It is the last of these three, namely, the training of NNs, which typically demands the greatest time, effort, and resource, and poses the greatest challenge to their development and deployment.

In previous exploratory studies (Abel and Spannowsky, 2021; Abel et al., 2021, 2022b), we showed that a NN can be trained by encoding it in a transverse Ising model on a quantum annealer (Lanting, 2017). The study also demonstrated that such an approach, utilizing quantum tunneling, can train a NN optimally, reliably, and quickly. Furthermore, the trained parameters can be extracted and used in a classical network for deployment. However, the restriction to a transverse Ising model as the Hamiltonian for quantum annealing greatly limits the expressivity of the NN. Essentially, this restriction means that the loss function can only be a quadratic function of binary variables. If a binary encoding for the free parameters of the NN is employed, the resulting loss function depends at most quadratically on the weights. Typical machine learning applications require more complex loss functions. This problem might be reduced by encoding higher-order polynomials using auxiliary variables and quadratic constraints, but this implies a large number of additional qubits. This is the cost of employing an Ising model. Essentially any polynomial ground-state Hamiltonian can be expressed by means of reducing it to quadratics (see Abel et al., 2022b for examples), but the number of qubits grows rapidly. Thus, to address these obstacles, this article proposes a universal AQC approach that can be used to train a neural network and extract the optimally trained network weights. The much wider variety of Hamiltonians that can be used within the universal AQC paradigm allows us to include correlations and non-linearities in our models, allowing adiabatic quantum training to be applied to larger and more expressive networks. In fact, the general form of the Hamiltonian allows one to encode, in principle, any given loss function.

We will present two techniques for performing the AQC: the “matrix method”, in which the system is expressed in terms of truncated Hilbert space components and the “Pauli-spin method”, in which it is expressed directly with Pauli-spin matrices. We apply these methods to simulated quantum-gate computers, showing the applicability of AQC training on near-term devices. Furthermore, we apply the “Pauli-spin method” to the training of both continuous neural networks and networks with discrete and binary weights. The latter usually rely on non-gradient-based optimization algorithms and are classically very difficult to treat.

In the burgeoning domain of computational intelligence, neural networks are heralded as the cornerstone of machine learning, particularly excelling in classification and regression tasks. Their influence permeates both everyday applications and advanced scientific research. Hence, being able to enhance their capabilities and streamline their training through the innovative lens of quantum computing is of considerable significance.

## 2 Challenges in training neural networks

This study initially discusses the difficulties one may encounter when training a neural network. In the training phase, the goal is to reach the global minimum of the so-called cost or loss function. However, the optimization landscape of neural networks often contains multiple local minima. This problem is only exacerbated if the network is small. Overall, in a space of high dimensionality, the most critical points are likely to be saddle points. Thus a gradient descent method is usually effective. Conversely, on the small neural networks, the global minimum can be much more difficult to find. Indeed, several other issues can arise during training in spite of the implementation of correct algorithm. Here, we briefly list these challenges and the typical approach that can be employed to deal with them in classical training:

- Slow progress, fluctuations or instability: tackled by optimizing the learning rate to either speed up or slow down convergence.
- Badly conditioned curvature: “ravines” in the landscape of values of the loss function in the free-parameter space imply that different directions require different learning rates to be optimal. That is, one must take larger steps in the directions in which the loss function varies slowly and smaller steps in those in which it changes rapidly. The Adam algorithm can address this by adapting learning rates individually for each parameter.
- Local Optima: the algorithm might become trapped in a local minimum of the loss function, since a small trial step in any direction will increase the loss. This is usually addressed by using random restarting points to explore every basin of attraction. It is also worth noting that in a large dimensionality parameter-space, the most critical points are saddle points. Thus, this is a problem that afflicts small NNs compared to large NNs.
- Weight degeneracy: the output of an NN is often invariant under some permutation of its parameters, but the optimal values of the parameters themselves are not. Indeed, this study demonstrates an example of this phenomenon in a later section. This is addressed by a random initialization of the weights and biases, which breaks the symmetry.
- Dead and Saturated Units: activations at the ends of their range cause plateaus in the loss-function landscape. Initializing biases with positive values can help avoid the problem, although it can also signal a redundancy in the network that one would like to reduce by pruning out redundant weights. This problem is often an indication that the NN can be “pruned”, that is, that part of the network is effectively redundant and can be removed. Unfortunately, determining precisely how a network can be pruned is difficult.

Following on from the last of these points, it is worth emphasizing that the paradigm of neural networks uses a set of *continuous* weights and biases on which a gradient descent can be performed. However, arguably, this causes great redundancy because, in many situations, a reasonable solution to the optimization of the network is, in principle, achievable with weights that are discrete or even binary (i.e., just “on” or “off”) if only we can find the correct discrete values.

To appreciate the redundancy that is inherent in continuous weights and biases, consider the example of a classification task when there are only two features. In principle, the classification curve can be written as the Taylor expansion of the level-curve of some function *z*(*x*_1_, *x*_2_) of the features *x*_1_ and *x*_2_. However, if this classification curve happens to be well approximated by a quadratic function, for example, then it would require only six continuous coefficients. In contrast, the neural networks would typically have many more continuous weights and biases. However, if we accept that these six continuous Taylor coefficients are well approximated if we know them to four binary places (i.e. to one part in 16), then only 24 binary weights taking values of 0 or 1 should *in principle* be able to describe the same classification curve. Because of this redundancy, there is indeed quite some interest in training discretely weighted networks and networks where both the weights and activation functions are binary (Courbariaux et al., 2015; Ding et al., 2019; Roth et al., 2020; Livochka and Shekhovtsov, 2021; Yuan and Agaian, 2023).

However, we can immediately appreciate that such a system of discrete weights is classically problematic precisely because it runs into both the “weight degeneracy” and the “dead and saturated units” problems mentioned in our list of challenges. Moreover the “local optima” problem is generic. Indeed it is for these reasons that the classical training of discretely weighted and binary systems requires special treatment (Roth et al., 2020; Livochka and Shekhovtsov, 2021).

## 3 Adiabatic quantum computing on gate quantum computers

Here, we discuss AQC and its general implementation on gate quantum computers.

Although it is interesting for the reasons outlined above to allow our eventual systems of interest to be relatively discrete in nature, it will be useful in establishing the basic principles first to consider systems of function of continuous variables. Thus in this section, we will focus on the specific task of finding all the global minima of a function *V*(*w*) of one variable in the interval *w* ∈ [0, 1] (We refer weights and biases using the variable *w*.). AQC is equivalent in this context to solving for the ground state in one-dimensional quantum mechanics with *w* corresponding to the single space dimension. Studying such familiar cases will allow us to confirm that our system behaves as expected.

**Figure 1:**
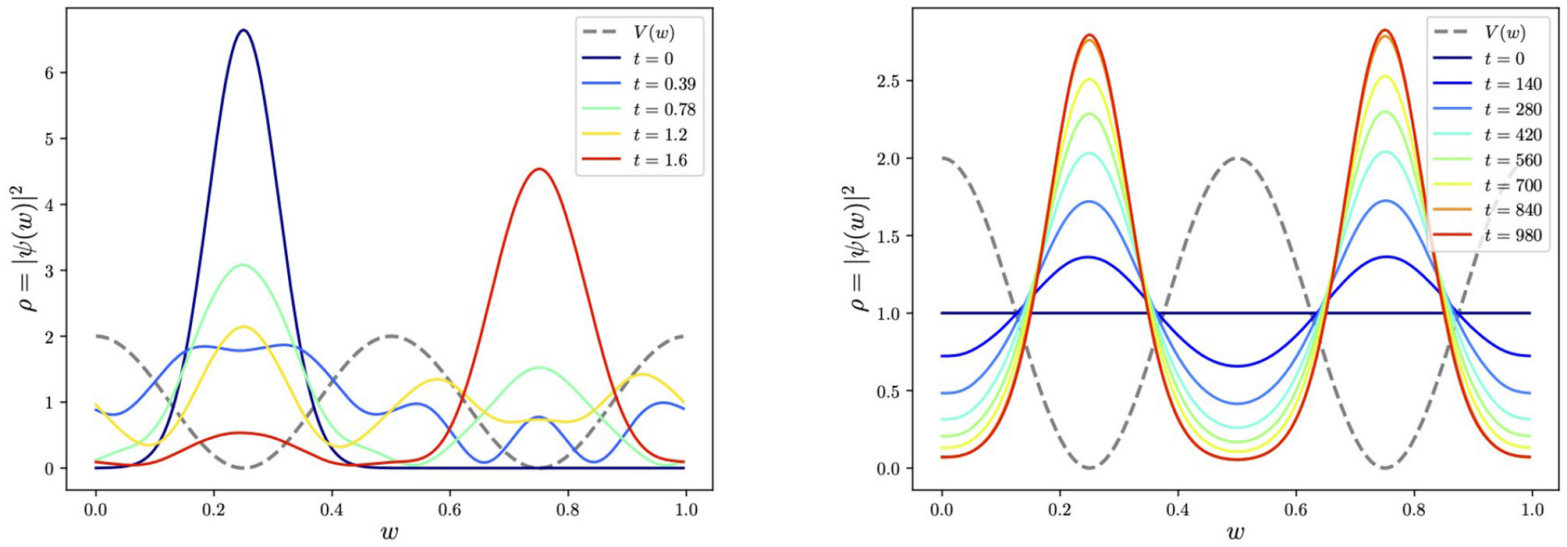
Tunneling vs. adiabatic evolution in the cosine potential, *V*(*w*) = 1 + cos(4π*w*), with a truncation at energy level 〈*w*|*n*〉 = *e*^2π*nwi*^, where *n* ∈ [−15, 15]. In the tunneling example, we take *m* = 10, while in the second adiabatic example, we take masses of *m* = 100 (equivalently *V* can be multiplied by 100) in order to get well-localized peaks in the ground state. The evolution time must be increased accordingly. The schedule function is taken to be simply linear, *s*(*t*) = *t*.

The first example we will look at is the following cosine potential which has two degenerate minima in the interval *w* ∈ [0, 1]:


(1) $$\begin{array}{l} V\left(w\right)\;=\;1+\cos\left(4\pi w\right)\;, \end{array}$$

which appears as the dashed line in Figure 1.

There are various ways in which one might wish to encode the problem of minimizing this potential. It is necessary to ensure that the chosen method is both effective and yields an advantage (in the sense that the difficulty does not scale exponentially with the problem size). Here, we shall consider two encoding methods, namely, the “matrix method” and the “Pauli-spin method”.

### 3.1 The matrix method

The Matrix method is the most direct: it entails evolving the wavefunction from some starting state using the Schrödinger Hamiltonian,


$$\begin{array}{l} Ĥ\;=\;\frac{{\hat{p}}^{2}}{2m}+V\left(ŵ\right)\;, \end{array}$$

where *m* is the mass and $\hat{p}$ is the momentum operator conjugated to the position operator ŵ, obeying $\left[ŵ,\hat{p}\right]=i\hbar$. We will henceforth take ℏ = 1. To proceed, we must cast Ĥ into its matrix form in a truncated Hilbert space. To do this, we adopt periodic boundary conditions and define a basis of eigenstates of the kinetic piece in the Hamiltonian working in the *w*-basis:


$$\begin{array}{l} \langle w|n\rangle \;=\;e^{2\pi inw}\;, \end{array}$$

where *n* ∈ ℤ labels the Fourier modes. The Hamiltonian matrix elements are then given by


$$\begin{array}{l} H_{n\ell }\;=\;\int_{0}^{1}\langle n|w\rangle \langle w|Ĥ|\ell \rangle dw\\ \;=\;\frac{4\pi^{2}n^{2}}{2m}+\tilde{V}\left(n-\ell \right)\;, \end{array}$$

where $\tilde{V}\left(n\right)=\int_{0}^{1}V\left(w\right)e^{-2\pi inw}dw$ is the Fourier transform of *V*(*w*), which we can easily calculate for any *n*, ℓ ∈ ℤ. Thus, we can in principle take the resulting matrix *H*_*nℓ*_ and use it to evolve the wavefunction ψ(*w, t*) from an initial state ψ(*w*, 0) = *c*_*n*_(0)〈*w*|*n*〉, using the Trotterized Schödinger evolution,


$$\begin{array}{l} c_{n}\left(t\right)\;=e^{-iH_{n\ell }t}c_{\ell }\left(0\right)\\ \;\approx \;{\left(e^{-iH_{n\ell }\delta t}\right)}^{t/\delta t}c_{\ell }\left(0\right)\;, \end{array}$$

where δ*t* is a small time step. Up to this point, everything is expressed in simple quantum mechanics. However, we wish to encode the wavefunction and its evolution in terms of qubits. This can be performed by truncating the Hilbert space to size 2^*N*^ with *n* ∈ [−2^*N*−1^, 2^*N*−1^]. This allows us to identify each index *n* with one of the 2^*N*^ possible eigenvalues of *N* tensored qubits. The simplest choice for this identification is to treat *n* like the computational-basis index: that is we associate the binary expression for each *n* with the eigenvalues of the *N* tensored binary operators


(2) $$\begin{array}{l} T=\frac{1}{2}\left(𝟙+Z\right)\;, \end{array}$$

where *Z* is the Pauli *Z*-spin matrix for each qubit. Thus, for example, |*n* = −2^*N*−1^〉 ≡ |000…000〉, |*n* = 0〉 ≡ |000…010〉, |*n* = 3〉 ≡ |110…010〉, and so forth.

To perform the time evolution, our 2^*N*^ × 2^*N*^ Hamiltonian matrix must then be accordingly decomposed into sums of tensor products of the Pauli-spin matrices, which act on the *N* tensored qubits, and then the evolution of the initial state Trotterized as above. To implement this step in the process, here and throughout, we will make extensive use of the Qibo package of programs, which allow fast evaluation of quantum circuits taking full advantage of hardware accelerators (Efthymiou et al., 2021, 2022, 2023; Robbiati et al., 2023). This package allows one to automate the decomposition step and implement the Trotterized time evolution induced by a symbolic Hamiltonian defined in terms of Pauli-spins, which is rendered as a quantum gate circuit. Moreover, simulation is feasible up to an order of 25 qubits.

As a warm-up exercise, it is interesting to consider an initial wavefunction localized in one of the minima and observe its tunneling to the other degenerate minimum. This is shown for exhibiting potential of Eq. (1) in the first panel in Figure 1, where we choose a Gaussian localized in the left minimum for the initial state. We perform the time evolution as a simulation using Qibo's “StateEvolution” module, which, as we discussed, produces and evolves the circuit corresponding to the symbolic Hamiltonian (Importantly, Qibo allows one to put the same Trotter evolution directly onto real machine.).

The wavefunction indeed tunnels to the second minimum, as expected. However, in this initial example, we can also see why quantum tunneling *per se* is not always beneficial for locating global minima. There is no energy dissipation in an idealized setting, thus the initial wavefunction never stops moving unless it is already in an energy eigenstate. It would, for example, be very hard to determine the global minimum if the minima were only slightly non-degenerate. This can be contrasted with dissipative systems such as those utilized in quantum annealers in Abel et al. (2022a,b), Criado and Spannowsky (2023), and Criado et al. (2023).

Hence to determine the true global minimum, we can use AQC as envisaged in Farhi et al. (2001, 2002, 2006). That is, we begin the system in the ground state of a trivial Hamiltonian Ĥ_0_ and adiabatically evolve the system to the complicated Hamiltonian of interest, Ĥ. As a function of time, the total Hamiltonian Ĥ_*A*_ for adiabatic evolution in the AQC paradigm takes the form


(3) $$\begin{array}{l} {Ĥ}_{A}\left(t\right)\;=\;\left(1-s\left(t\right)\right){Ĥ}_{0}+s\left(t\right)Ĥ\;, \end{array}$$

where *s*(*t*) is the so-called schedule function with *s*(0) = 0 and *s*(*t*_final_) = 1. If the evolution is sufficiently adiabatic, the system will always remain in the ground state. The result is the desired ground state of the complicated Hamiltonian of interest. For the present example, we can take Ĥ_0_ to be the purely kinetic Hamiltonian with *V* = 0, for which the *n* = 0 state, 〈*w*|ψ〉 = 〈*w*|000…010〉 = 1, is trivially the ground state solution.

We performed the adiabatic evolution within Qibo using models.AdiabaticEvolution, with the schedule taken to be linear for simplicity, *s* = *t*/*t*_final_. The resulting evolution is shown in the second panel in Figure 1. Notably, the complicated Hamiltonian's eventual ground state function is time-independent as it should be and correctly responds to the two minima degenerately. Thus, for locating the global minima, the mass (or, more generally, the kinetic to potential terms ratio in Ĥ) plays an important role. The higher the mass is relative to *V*(*w*), the sharper the peak around the global minima. This is, of course, to be expected because approximating the potential around each minimum, *w*_min_, as a simple harmonic oscillator (SHO), the wavefunction is of the form


$$\begin{array}{l} \rho_{0}\left(w\right)=|\psi_{0}{|}^{2}\;\approx \;{\left(m\right)}^{1/4}e^{-4\pi \sqrt{m}{\left(w-w_{\text{min}}\right)}^{2}}\;. \end{array}$$

**Figure 2:**
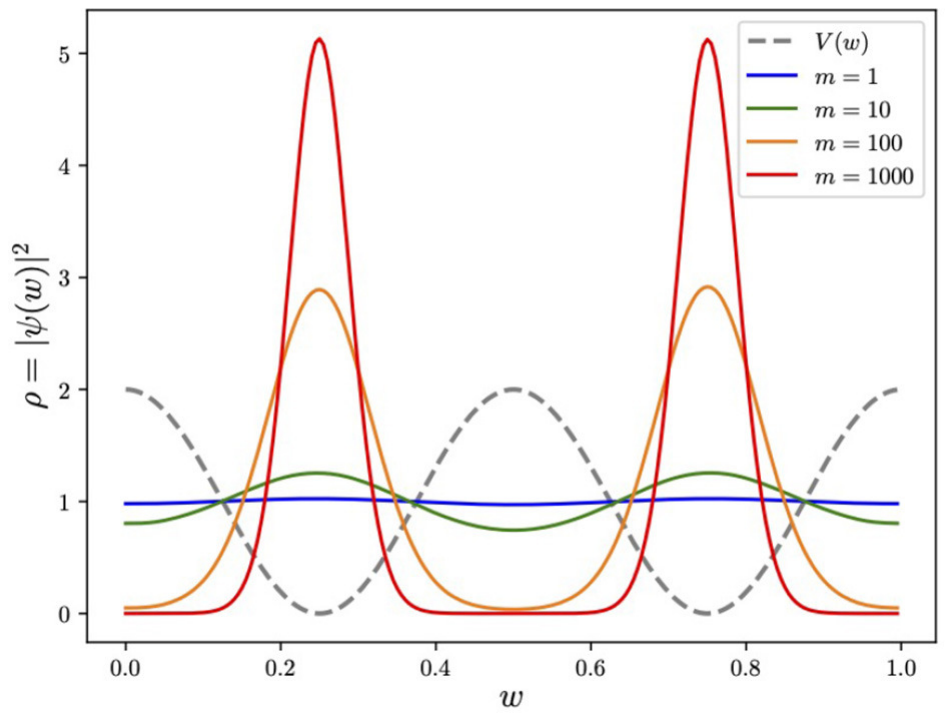
The effect of mass on the ground state. Around each minimum, the ground state approximates the Gaussian ground state of the SHO with *V*(*w*) = 8π^2^*w*^2^, namely, $\rho_{0}\left(w\right)=|\psi_{0}{|}^{2}\approx {\left(m\right)}^{1/4}e^{-4\pi \sqrt{m}{\left(w-w_{\text{min}}\right)}^{2}}$ (normalized such that each of the two peaks contributes 1/2).

We show this dependence explicitly in Figure 2, which displays the expected *m*^1/4^ behavior in the amplitude of the peaks. This feature will be important in later discussions.

**Figure 3:**
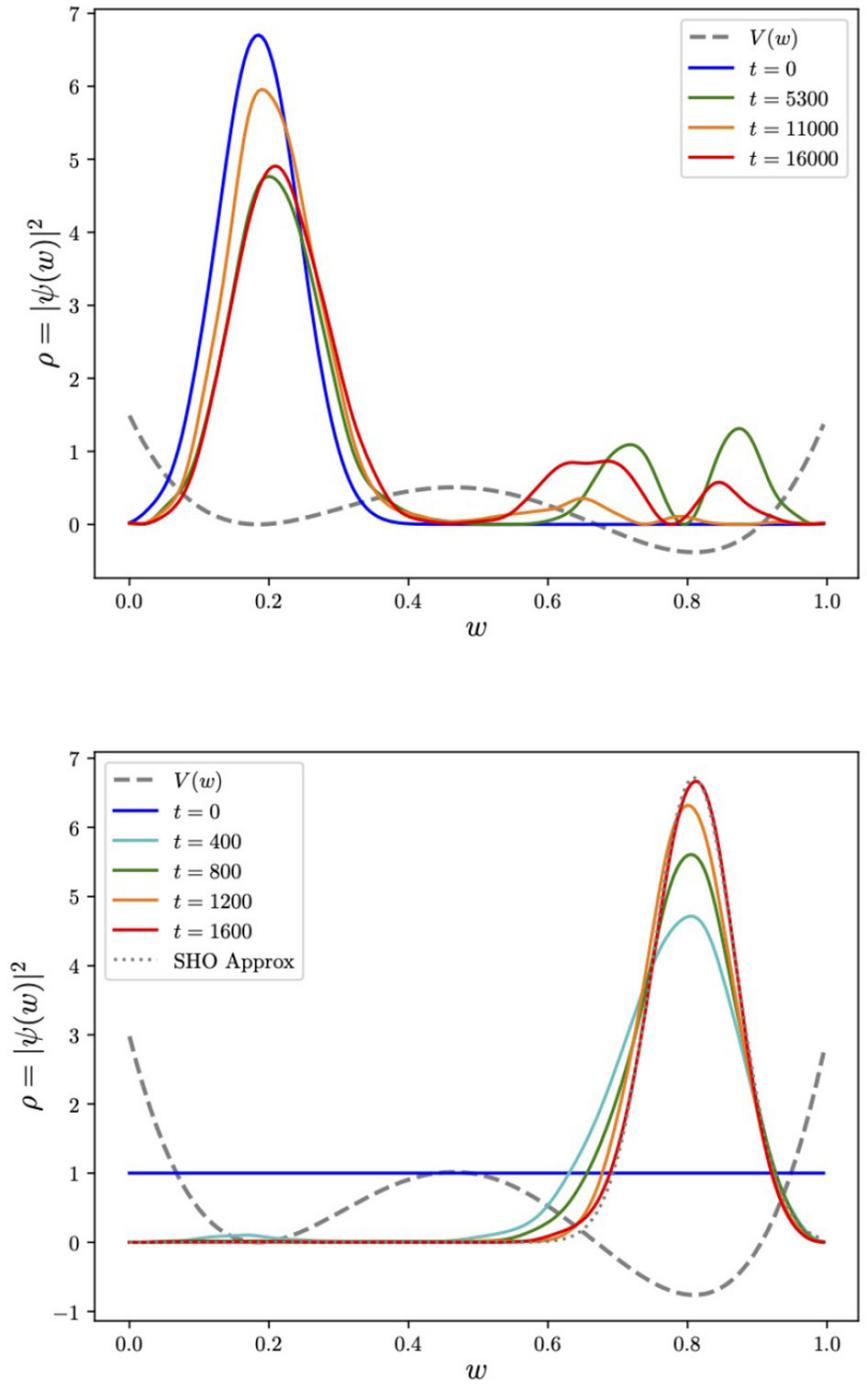
Tunneling vs. adiabatically evolving the ground state in a quartic potential. Here, for tunneling, the initial wavefunction is chosen to be the ground state of the approximate SHO potential around the false minimum (with λ = 4, *m* = 100). For the adiabatic evolution, we take λ = 8, *m* = 200 to ensure a localized peak at the origin. We also show (overlaid dotted line) the ground state of the SHO approximation, which is obtained by expanding around the global minimum at *w* = 0.8.

It is instructive and useful for our later discussion to perform the same kind of comparison in a polynomial potential with a metastable minimum, where the system can be trapped. A simple case is the following quartic potential:


$$\begin{array}{l} V\left(w\right)\;=\;\lambda \left(18w^{4}-35w^{3}+22w^{2}-5w+0.372573\right)\;, \end{array}$$

where λ is an overall factor to scale the potential. The potential is shown as the gray dashed line in Figure 3. To examine tunneling, we begin the system in the approximate ground state of the metastable minimum at *w*_+_ = 0.1848. Expanding around this point, we find an approximate SHO potential with $V\left(w\right)\approx \lambda \left(0.372573+2\pi {\left(w-w_{+}\right)}^{2}\right)$ (and hence SHO parameters $m\Omega =2\sqrt{\lambda \pi m}$). Thus to demonstrate tunneling, we begin the system in the Gaussian ground state,


$$\psi_{0}\left(w\right)\;=\;{\left(4m/\pi \right)}^{1/8}e^{-\sqrt{\lambda \pi m}{\left(w-w_{\text{min}}\right)}^{2}}\;.$$

**Figure 4:**
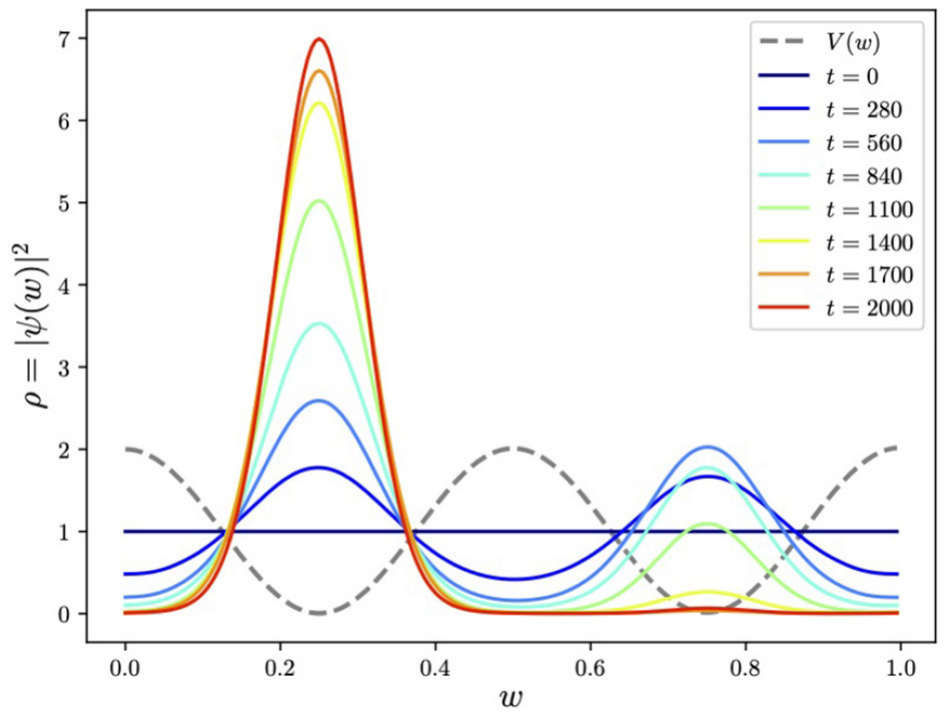
AQC for the exact same system as in Figure 1 but with not quite degenerate minima (the energetic difference between the two minima being Δ*V*_min_ ≈ 0.01). During the evolution the ground state ultimately selects the true minimum provided the process remains adiabatic.

The subsequent evolution is shown in the first panel in Figure 3. We also notice that tunneling does not help locate minima without some element of dissipation. Indeed the wavefunction either oscillates wildly between the minima on longer timescales or remains relatively stuck: it is quite challenging to control the behavior, which depends sensitively on the choice of both λ and *m*. This can be contrasted with AQC which correctly reproduces the ground state in the second panel. This only selects the true global minimum even when the two minima are almost degenerate. As an example of the latter, we show in Figure 4 the evolution of the cosine potential (performed using the “matrix method”) with a tiny linear term Δ*V*(*w*) = ϵ*w*, where ϵ = 0.02, which causes non-degeneracy in the two minima. Even though the two minima are imperceptibly non-degenerate, the adiabatic process ultimately finds the true global minimum. The behavior is quite striking because it is initially degenerate, and only toward the end of the process, the true minimum is selected. As for the cosine potential, the global minimum can be more precisely located by increasing the mass or increasing the parameter λ, subject to the constraint that the Trotterization should remain a good approximation (i.e., |*H*|δ*t* ≪ 1). However, in the present context, the most important aspect of this example is that we can see that AQC completely avoids the “Badly Conditioned Curvature” problem mentioned in our list of challenges in Section 2.

We should, for completeness, attach a caveat to this picture: the oscillation back and forth that we can observe in the tunneling solutions is partly due to the fact that the systems we consider in these illustrative examples are only one-dimensional and periodic. Quantum tunneling in many physical systems of interest (for example, phase transitions in cosmology) is higher dimensional and takes place in non-compact volumes. In such situations, the tunneling process is one-way because there is a large degenerate volume of global minima: excess energy after tunneling is dissipated in dynamics, for example, in accelerating bubble walls.

### 3.2 The Pauli-spin method

Despite the straightforward nature of the matrix method for adiabatic evolution, it is not the most convenient approach for training NNs because the matrix *H*_ℓ*n*_ would grow exponentially with the number of variables (i.e. weights) in the system, requiring us to store a 2^*N*^ × 2^*N*^ matrix in general. Therefore, it is typically more efficient (we will make a more detailed comparison of the relative efficiencies later in Subsection 4.4) to use the “Pauli-spin method”. In this method, the variables and hence the Hamiltonian are encoded in a binary fashion in the eigenvalues of Pauli-spins.

That is, we assign bin values for the variables themselves instead of the wavefunction by defining the binary *T* operators as in Eq. (2). For example, in the single variable case, we encode *w* discretely as a fractional binary composed of *N* of the binary spins, *T*_ℓ_. Hence, the operator corresponding to *w* is given by


(4) $$\begin{array}{l} ŵ\;=\;2^{-N}\overset{N-1}{\underset{\ell =0}{\sum }}2^{\ell }T_{\ell }\;. \end{array}$$

The above encoding yields binned values for possible measurements of the variable, $\langle ŵ\rangle \in \left\{w_{r}\right\}=\left\{0,\frac{1}{2^{N}},\frac{2}{2^{N}}\ldots ,1-\frac{1}{2^{N}}\right\}$. Thus, any particular state |ψ〉 is defined as


$$\begin{array}{l} |\psi \rangle \;=\;\underset{r}{\sum }|w_{r}\rangle \langle w_{r}|\psi \rangle \;, \end{array}$$

with *r* = 0…2^*N*−1^ labeling the possible bin values *w*_*r*_ and with $\rho \left(w_{r}\right)=|\langle w_{r}|\psi \rangle {|}^{2}$ yielding the probability for measuring the state in that particular bin. Primarily, this replaces the momentum truncation with a direct variable discretization.

This is the general structure for encoding variables. How should we now go about constructing the adiabatic evolution? For the target Hamiltonian Ĥ, the main aspect to note is that in this discretized variable formulation of the problem, the momentum and hence the kinetic ${\hat{p}}^{2}/2m$ terms would be hard to encode (such terms would have to be encoded by the finite difference which would greatly complicate the Hamiltonian). However, we also note that the kinetic terms in the Hamiltonian did not significantly contribute to locating the global minimum of *V*(*w*); their primary role it to provide spread in the profile of the eventual ground state. Indeed from Figure 2, it is clear that if we were to take the limit *m* → ∞ keeping *V*(*w*) unchanged, then the final wavefunction would be a spike at the global minimum, which would for optimization be virtually the ideal outcome. Thus, to determine the global minimum of a potential *V*(*w*), we may delete the kinetic terms and set


(5) $$\begin{array}{l} Ĥ\;=\;V\left(ŵ\right)\;, \end{array}$$

where the operator ŵ is to be replaced by its encoding in terms of *Z*_ℓ_ spins given in Eq. (4). It should be noted that, unlike the matrix approach, we are now constrained to consider polynomial potentials. In some of the applications we consider below, in Section 4, the type of potential required to train a given model is precisely a polynomial. In others, however, it is a non-polynomial function involving the composition of linear transformations and non-polynomial activation functions. As discussed in Section 4, this does not represent a problem in practice, since any bounded continuous function in a bounded domain may be approximated arbitrarily well by a polynomial function. For a given application, one can thus first find a suitable polynomial approximation and then apply the method to it. Moreover, a modest amount of reduction can be performed on the Hamiltonian. For example, upon expanding the polynomial $\hat{V}$, we may find powers of Pauli matrices that can be reduced using *T*_ℓ_*T*_ℓ_ = *T*_ℓ_ (These reductions are more significant when fewer qubits are used to define each ŵ).

To play the role of the trivial Hamiltonian in the adiabatic evolution, Ĥ_0_, we can use the commonly adopted transverse AQC choice,


(6) $$\begin{array}{l} {Ĥ}_{0}\;=\;\frac{1}{2}\overset{N-1}{\underset{\ell =0}{\sum }}\left(𝟙-X_{\ell }\right)\;, \end{array}$$

where *X*_ℓ_ is the *X* Pauli-spin matrix for the ℓ'th qubit. It can be easily noted that $\frac{1}{2^{N/2}}\prod_{\ell }$(|0〉*_ℓ_* + |1〉*_ℓ_*) is the ground state of this Hamiltonian (because *X*(|0〉+|1〉) = (|0〉+|1〉)). On expansion, we see that this is the state with degenerate probability in each *w* bin, which has 〈Ĥ_0_〉 = 0.

**Figure 5:**
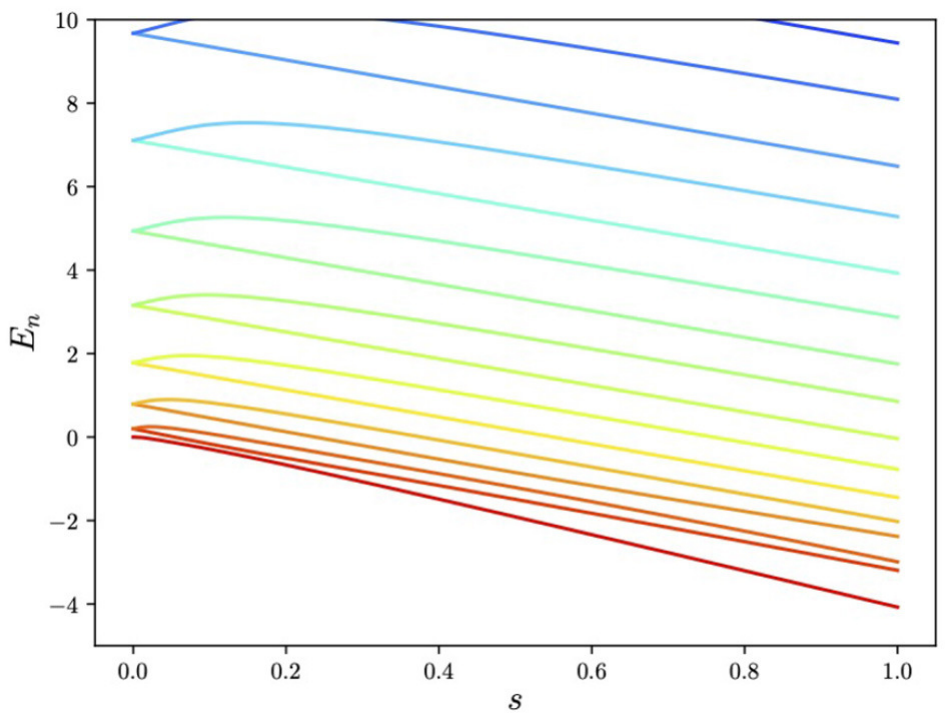
Energies released during the adiabatic evolution in Figure 3 showing the isolated ground-state energy.

**Figure 6:**
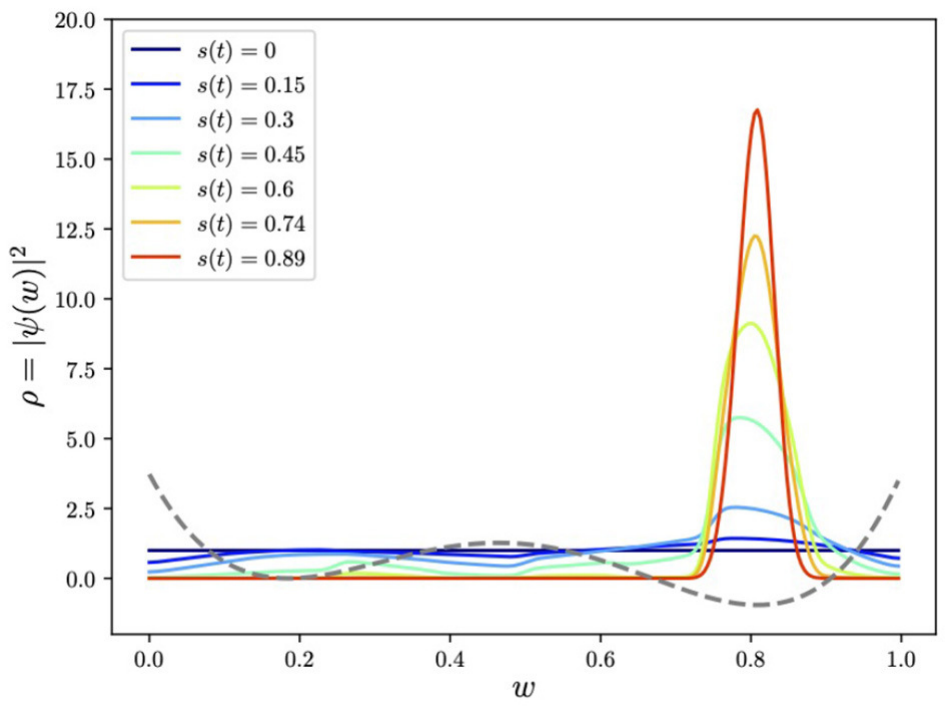
Adiabatically evolving to find the global minimum of the quartic potential using a Pauli-spin encoding of *w*. Here we show the evolving smoothed histogram of the ground state ρ(*w*) ≡ |ψ(*w*)|^2^ with *w* encoded in *N* = 7 qubits.

Finally, we put these two Hamiltonian components, namely, Ĥ_0_ of Eq. (6) and Ĥ of Eq. (5), into the adiabatic evolution equation in Eq. (3), and the system is evolved from the initial Ĥ_0_ ground state using the Trotterized circuit generated by Qibo. The result for the quartic potential is shown in Figures 5, 6. As expected, it is highly peaked around the global minimum.

## 4 The training of neural networks

### 4.1 The general method

In this section, we will demonstrate that the AQC optimization algorithm outlined in the previous section can be used to train machine-learning models, where we will now replace the single ŵ operator with a large number of weights and biases. We will focus on the supervised learning framework, which aims to find a function *Y*(*x*) that approximately reproduces a given set of outputs *y*_*a*_ from a given set of inputs *x*_*i*_. A classification problem is when the outputs, called labels in that context, take values in a small discrete set. Otherwise, the problem becomes general non-linear regression.

A machine learning model is a family of functions from which the optimal *Y*(*x*) function for the available data has to be selected. The process of finding this optimal function is known as training, and it is typically done by minimizing a *loss function* L, which measures the deviation of the predictions *Y*(*x*_*a*_) from the labels *y*_*a*_. For example, one may define it as the mean squared error


$$\begin{array}{l} L=\frac{1}{N}\overset{N}{\underset{a=1}{\sum }}{\left(Y\left(x_{a}\right)-y_{a}\right)}^{2}. \end{array}$$

Some of the most versatile models in this setting are neural networks, which are constructed as the composition of layers *L*_*k*_(*z*), with each layer given by an affine transformation followed by the element-wise application of a non-linear function *f*_*k*_:


$$\begin{array}{l} Y\left(x\right)=L_{n}\left(\cdots L_{1}\left(x\right)\right),\\ L_{k}\left(z\right)=f_{k}\left(\underset{j}{\sum }w_{ij}^{\left(k\right)}z_{j}+b_{i}^{\left(a\right)}\right). \end{array}$$

The parameters *w* and *b* are known as the weights and biases, and the functions denoted by *f* are called the activation functions.

Various classical algorithms have been developed to optimize the loss function L. Most of them are local optimization methods, in which the weights and biases are updated iteratively in minor increases. A common problem that these algorithms can only partially address is that they can become trapped in local minima for a non-convex loss function. Thus, quantum algorithms are capable of preventing this problem by directly tunneling or adiabatically evolving toward the global minimum, which work qualitatively differently from classical gradient-based optimization methods and prevent these problems.

In Section 3, we outlined two general methods for minimizing arbitrary functions, namely, the “matrix method” and the “Pauli-spin method”. We shall now apply the Pauli-spin method to minimize the loss as a function of the free parameters of the neural networks, which are weights and biases.

First, we provide some general remarks on the advantages and disadvantages of the two methods in the neural-network context. As we noted, the Pauli-spin method enables an efficient representation of the Hamiltonian in terms of Pauli matrices at the price of approximating the function through polynomials. In the context of neural networks, this implies that the activation function must be approximated by a polynomial, such that the loss function becomes a polynomial in spin matrices of degree given by the number of layers and the degree of the activation function. Consequently, one only needs to store the non-vanishing coefficients of this polynomial. This can be a significant advantage over matrix encoding. The effects of the polynomial approximation can be made arbitrarily small because any well-behaved activation function can be approximated arbitrarily well by a polynomial in a bounded domain. The range of values of the inputs to each activation is bounded and known in advance, given the range of values of the inputs *x* and the binary-encoded parameters *w* and *b*. The disadvantage of the “Pauli-spin method” is that the nested non-polynomial activation functions result in a large gate depth. We will make more quantitative comparisons of the methods later in Subsection 4.4.

### 4.2 Toy example

For concreteness, we will focus on a toy example, although the method can be used to train any other neural networks. Our neural network has two layers, the first mapping 2D points to 2D points and the second mapping 2D points to numbers. We take activation functions to be $f_{1}\left(x\right)=x^{2}$ and *f*_2_(*x*) = *x* and the biases $b_{i}^{\left(1\right)}=0$ and *b*^(2)^ = −1. The output is then given by


$$\begin{array}{l} Y\left(x\right)=\left(\begin{array}{cc} w_{1}^{\left(2\right)} & w_{2}^{\left(2\right)} \end{array}\right){\left[\left(\begin{array}{cc} w_{11}^{\left(1\right)} & w_{12}^{\left(1\right)}\\ w_{21}^{\left(1\right)} & w_{22}^{\left(1\right)} \end{array}\right)\left(\begin{array}{c} x_{1}\\ x_{2} \end{array}\right)\right]}^{2}-1, \end{array}$$

where the square is to be understood as the element-wise square function applied to a 2-vector. We use the Pauli-spin method, with one qubit per parameter only. This leads to a system with a total of 6 qubits, which allow us to simulate it on a small classical computer using Qibo as described in the previous section.

We will use this network to perform a binary classification task, predicting a point to be signal if *Y*(*x*) ≥ 0 and background otherwise. We therefore call the *Y*(*x*) = 0 contours the decision boundary. The simple structure we have chosen allows for several shapes of the decision boundary, from which the optimal one is to be selected by the adiabatic computation.

The two datasets we consider are a set of 1000 randomly chosen 2D points, with uniform distribution in the square [−1, 1] × [−1, 1]. In the first one, which we call the circle dataset, these 2D points are labeled as *y* = 1 (signal) if *x*^2^ + *y*^2^ > 1/2 and *y* = −1 (background) otherwise. The optimal decision boundary for the circle dataset is thus the circle *x*^2^ + *y*^2^ = 1/2, which our toy neural network can achieve. In the second dataset, which we call the band dataset, they are labeled *y* = 2 (signal) with probability given by max[1, (*x* + *y*)^2^] and with *y* = −2 otherwise. We make this choice so that the data is not perfectly separable, but our neural network can achieve the lowest value of the loss function when it generates a decision boundary of 2(*x*^2^ + *y*^2^) = 1.

**Figure 7:**
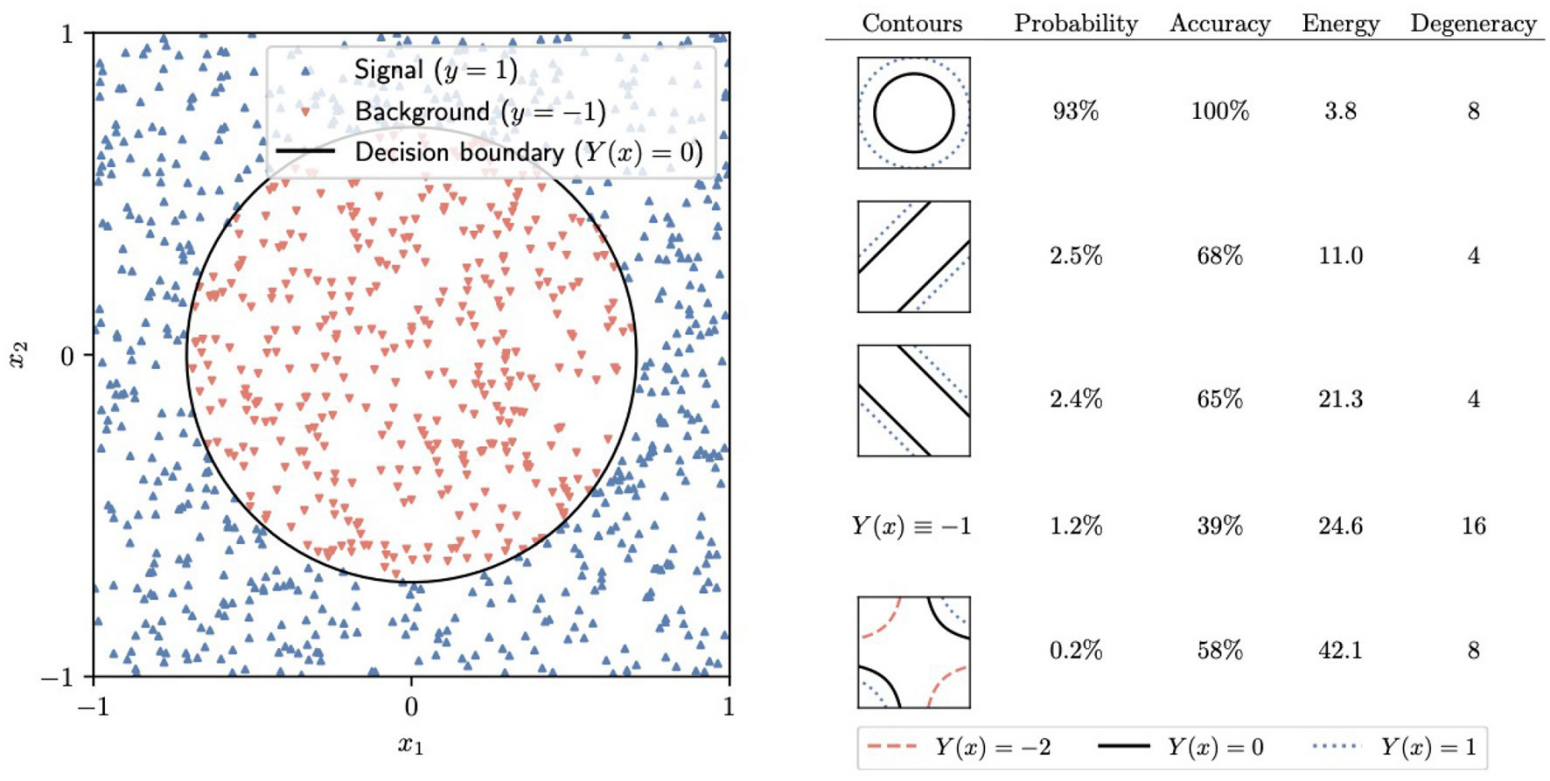
**Left:** The circle dataset and the corresponding decision boundary generated by the most probable final state after adiabatic evolution and measurement. **Right:** A summary of some of the potential outcomes of the final measurement, including the corresponding *Y*(*x*)= constant contours, the probability of measuring each of them, their energy, and the degeneracy [the number of equivalent states that generate the same *Y*(*x*) function].

**Figure 8:**
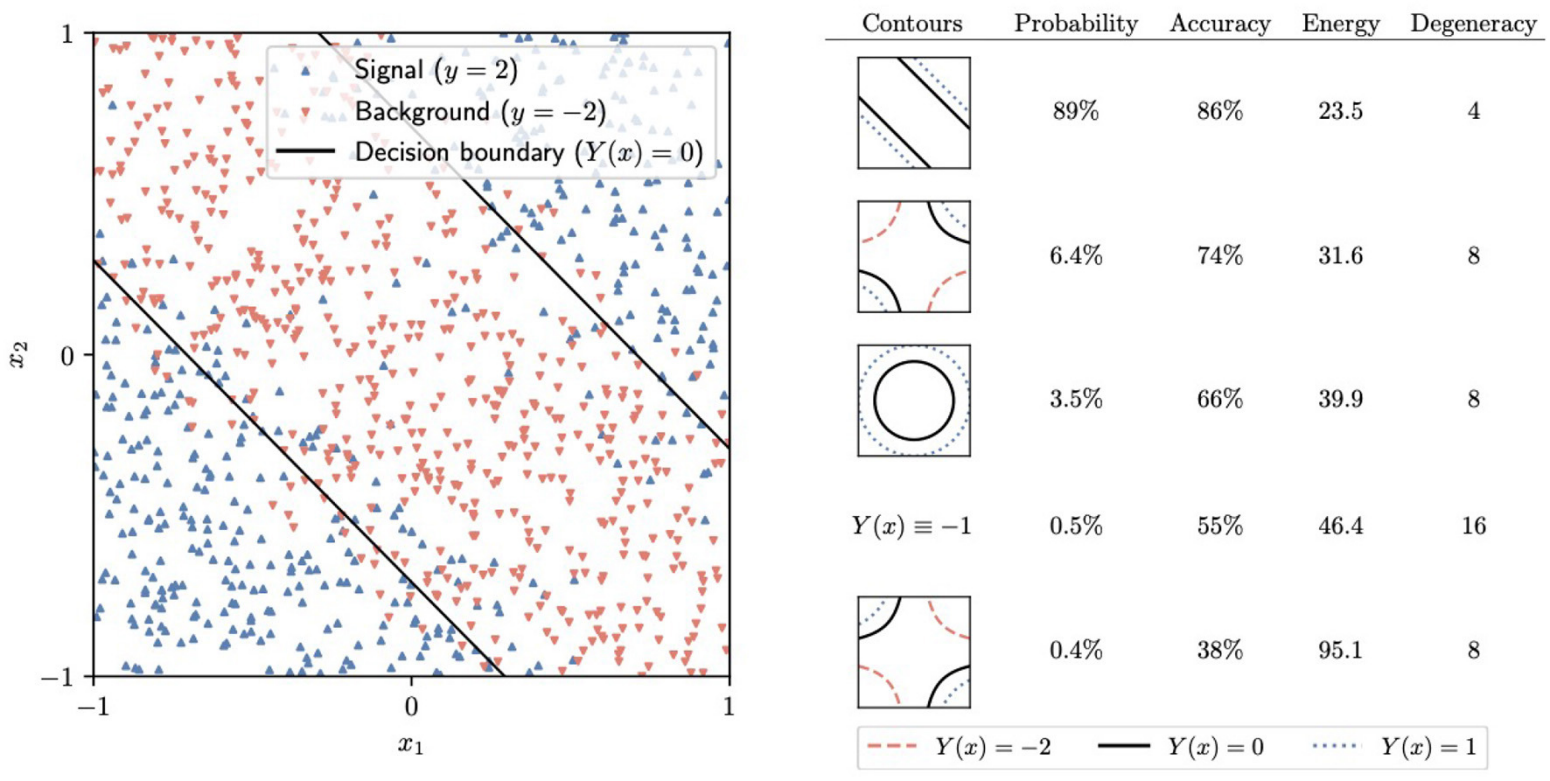
**Left:** The band dataset and the corresponding decision boundary generated by the most probable final state after adiabatic evolution and measurement. **Right:** A summary of some of the potential outcomes of the final measurement, including the corresponding *Y*(*x*)= constant contours, the probability of measuring each of them, their energy, and the degeneracy [the number of equivalent states that generate the same *Y*(*x*) function].

To train the neural networks, we generate the target Hamiltonian Ĥ by replacing each weight in the loss function L by a *Z* Pauli matrix. The initial trivial Hamiltonian Ĥ_0_ is given by Eq. (6). We use Qibo to simulate the adiabatic time evolution in 10 steps from *t* = 0 to *t* = 10, with a linear schedule *s*(*t*) = *t*/10. The final state consists of a superposition of different computational-basis states. In a real-world device, one would measure all of the *Z*_ℓ_ to obtain the classical values of the weights in the network. Our simulation shows that the correct contour for the circle dataset, displayed on the left in Figure 7, is the most likely outcome of this measurement, with a 93% probability. Similarly, the most likely outcome for the band dataset, with 89% probability, is the optimal contour, shown on the left in Figure 8. In practice, performing a low number of AQC runs and selecting the final state with the least energy is a viable strategy.

It should be noted that, similar to most neural networks, the one we are considering has multiple symmetries because different possible values of the weights give rise to the same function *Y*(*x*). An example of such a symmetry consists of flipping both $w_{11}^{\left(1\right)}\to -w_{11}^{\left(1\right)}$ and $w_{12}^{\left(1\right)}\to -w_{12}^{\left(1\right)}$. Two states related to these symmetries must have the same energy under the target Hamiltonian Ĥ. On the right side of Figures 7, 8, we have collected the total probability of measuring any of the states leading to each of the most likely *Y*(*x*) functions.

One of the consequences of these symmetries is that the minima of the loss function are degenerate, and therefore, we are morally in the degenerate minima situation of Section 3. In the classical setting, the random initial seed of the optimization algorithm would select one of the degenerate minima. However, guided by the discussion in section 3, it is clear that quantum training leads to a different situation, in which the final quantum state is in a superposition of the degenerate minima, all of which have equal probability. It is thus the final measurement that plays the role of randomly selecting one of the minima. Moreover, it is clear that, generally, one cannot take many measurements and use the expectation values of the weights for the classical values because this would incorrectly average over these degenerate possibilities.

### 4.3 Binary neural networks

The limited number of qubits available in current quantum computers makes it more interesting to consider them for training smaller machine-learning models. A valuable class of such models, with many real-world applications, are binary neural networks (Qin et al., 2020; Yuan and Agaian, 2023). These are neural networks in which the weights can only take the values 0 or 1, the biases are set to 0, and the activation functions are given by


$$\begin{array}{l} f\left(\overset{n}{\underset{j=1}{\sum }}w_{ij}x_{j}\right)\;=\;\Theta \left(\overset{n}{\underset{j=1}{\sum }}w_{ij}x_{j}-\frac{n}{2}\right), \end{array}$$

where the *w*_*ij*_ and the *x*_*j*_ are the weights and inputs of the corresponding layer and Θ is the Heaviside step function. The *i*th output of each layer is 1 if at least half of the terms *w*_*ij*_*x*_*j*_ are 1, and zero otherwise.

Binary neural networks promise several advantages. In particular, using activation functions with 0 or 1 gives a naturally “pruned” network. This leads to enhancement of both explainability and interpretability as defined and discussed in Qin et al. (2020) and Leblanc and Germain (2022). Binary neural networks can be directly encoded in quantum computers without any loss of expressiveness that we encountered with a polynomial approximation of activation functions and with the discretization of continuous weights. The model trained in a quantum device can be *exactly the same* as the one implemented in a classical computer. This can be easily observed by noting that the binary 0/1 weights can be encoded using the binary *T*_ℓ_ operators constructed from Pauli *Z*_ℓ_ matrices via Eq. (2). The activation functions can be viewed as polynomials in the *T*_ℓ_'s, through the identity


(7) $$\begin{array}{l} f\left(T_{i}\right)=\overset{\left\lfloor n/2\right\rfloor }{\underset{m=0}{\sum }}\underset{i_{1}<\ldots <i_{m}}{\sum }\underset{j\neq i_{1},\ldots ,i_{m}}{\prod }T_{j}\underset{k=i_{1},\ldots ,i_{m}}{\prod }{\bar{T}}_{k}, \end{array}$$

where $\bar{T}=1-T$. For example,


$$\begin{array}{l} f\left(T_{1},T_{2},T_{3}\right)\;=\;T_{1}T_{2}T_{3}+T_{1}T_{2}{\bar{T}}_{3}\\ \;+T_{1}{\bar{T}}_{2}T_{3}+{\bar{T}}_{1}T_{2}T_{3}\;. \end{array}$$

The discrete nature of binary neural networks makes them even more difficult to train with conventional classical methods, which are, as we have seen, typically based on the gradient descent method (Qin et al., 2020; Yuan and Agaian, 2023). In principle, adiabatic quantum training completely avoids this issue, as it can be performed using the same procedure as we outlined above for quasi-continuous neural networks.

Since the outputs are binary (assuming that the labels *y* are binary as well), one can use a simpler linear loss function,


$$\begin{array}{l} L\;=\;\underset{a}{\sum }{\left(-1\right)}^{y_{a}}Y\left(x_{a}\right)\;. \end{array}$$

With such a loss function those points *x*_*a*_ with either label, *y*_*a*_ = 0 or *y*_*a*_ = 1, are penalized by one unit in the loss function if there is an incorrect prediction, *Y*(*x*_*a*_) ≠ *y*_*a*_ (That is {*y*_*a*_, *Y*} = {0, 1} is incorrect and contributes ΔL = 1 vs. {*y*_*a*_, *Y*} = {0, 0} which contributes ΔL = 0. Likewise, {*y*_*a*_, *Y*} = {1, 1} is correct and contributes ΔL = −1 vs. {*y*_*a*_, *Y*} = {1, 0} contributes ΔL = 0.).

**Figure 9:**
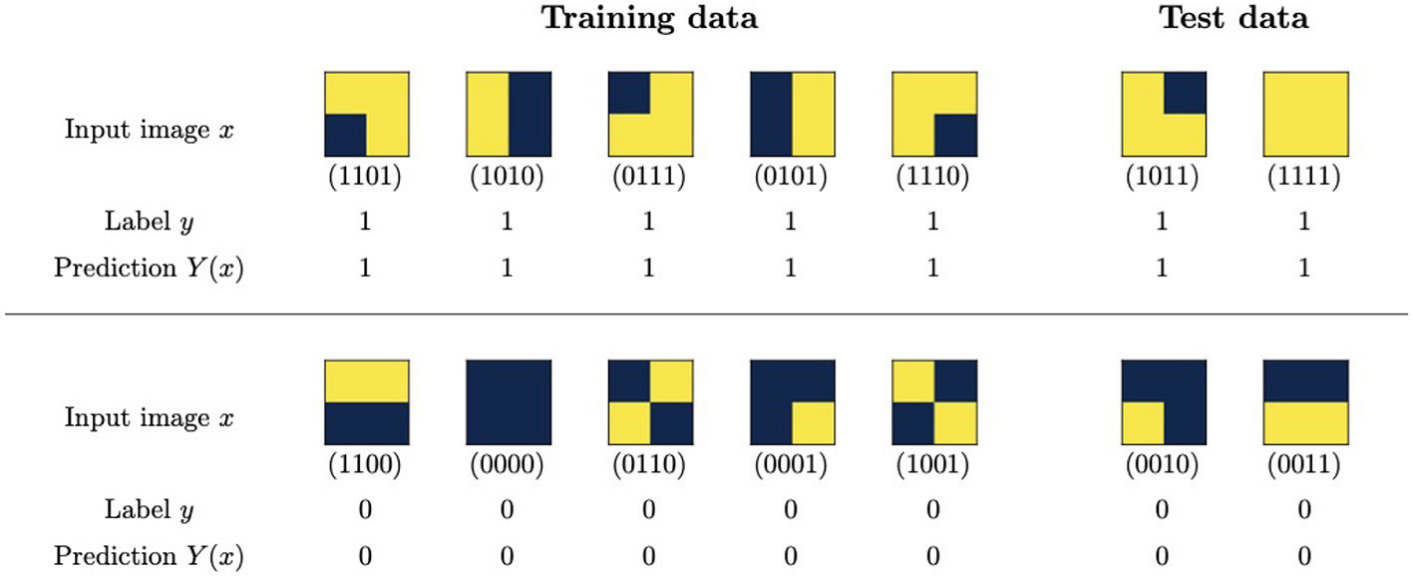
Dataset and predictions from an adiabatically-trained binary neural network. The predictions are generated using the weights determined by the most likely outcome after the final measurement.

To test this approach, we prepare a dataset of images with 2 × 2 binary pixels, labeling them with *y* = 1 (signal) if there are two pixels set to 1, one directly above the other, and *y* = 0 (background) otherwise. We select seven signal and seven background samples to balance the dataset. We then split the dataset into 5 (signal) + 5 (background) training images to be included in the loss function and 2 + 2 test images to check the generalization properties of the trained model. The selection and splitting are performed randomly from the 16 possible binary images. The resulting train/test datasets are displayed in Figure 9.

For the binary neural networks, we choose one with two layers, with the first having four inputs and two outputs and the second having two inputs and one output. The total number of weights, which are in one-to-one correspondence with the qubits, is 10.

To train the network, we use Qibo to simulate an adiabatic computation as described in the previous section, with Ĥ is now determined by substituting the expression for the weights in terms of qubits into the loss function L and the polynomial representation of the step function in Eq. (7). The predictions generated by the most likely weights after the final measurement are shown in Figure 9. They are 100% accurate in both the training and the test sets. The probability of obtaining these perfectly accurate weights in the final measurement is 18%. To assess the efficiency of the training, this can be compared with the portion of the space of weights that generates such predictions, which is 0.2%.

**Figure 10:**
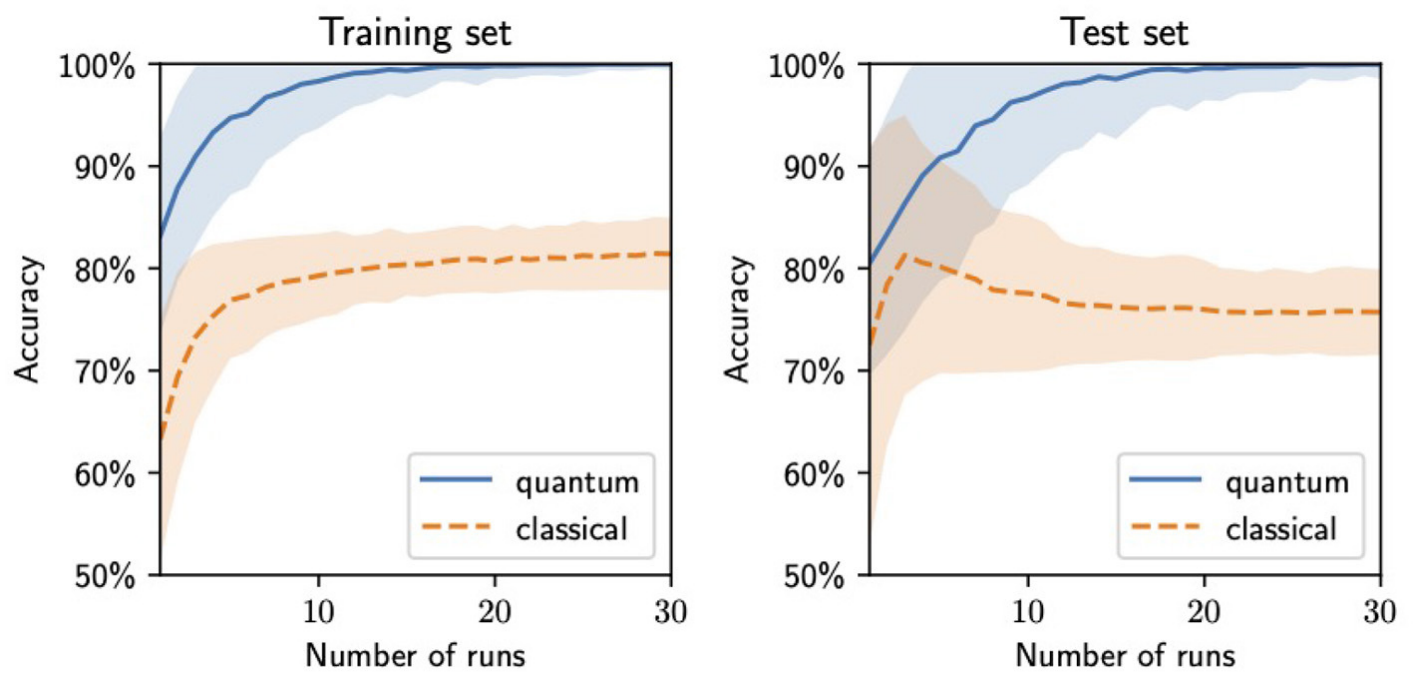
Binary neural network accuracies in the training **(left)** and the test **(right)** sets from the weights generated by running the training several times and selecting those with the best performance in the training set as a function of the number of runs. The central lines indicate the average accuracy value, and the bands show the standard deviation interval (both computed by repeating the process 1,000 times for each value of the number of runs).

Since the best values of the weights are obtained with the highest probability but not with certainty, it is profitable to perform several runs of the adiabatic computation and select the one that results in the highest accuracy in the training set. In Figure 10, we show how the accuracy of the trained network on both the training and the test sets improves with the number of runs. To obtain it, we generate a pool of 1000 sets of trained weights, with distribution given by the final state of the adiabatic evolution, before the final measurement. For each value of the number of runs *n* shown in Figure 10, we select *n* sets of weights from the pool and pick the maximum accuracy. This process is repeated 1,000 times, and the average and standard deviation of the resulting accuracies are displayed in the figure.

The nature of these highly accurate results may seem unusual in comparison with those typical for larger datasets. However, for a small dataset, it is reasonable that it can be exactly described by a simple model. In fact, the dataset was constructed with the simple rule that *y* = 1 if and only if there are two “on” pixels, one directly above the other. When a 100% accuracy is achieved in the training set, two options are obtained: either the model has learned this rule or it has overfitted, learning to identify only the data points in the training set but unable to generalize. If this was the case, one would expect roughly random results on the test set, giving approximately 50% accuracy. Instead, as shown in Figure 10, the model gives similar accuracies in the training and test sets for the same number of runs, which indicates no overfitting and points to the model having learned the underlying rule.

We compare the results of the quantum training with those of a classical analog trained using the Adam gradient descent algorithm. To construct this analog, we replace the step functions with sigmoids, replace the binary weights with continuous ones, and add a penalty term to the loss function of the form *w*^2^(*w* − 1)^2^ for every weight. The effect of this penalty term is to drive the weights to values 0 or 1. The classical values displayed in Figure 10 correspond to the same process as for the quantum ones described above, using a pool of 1,000 values generated through 1,000 classical training runs.

The quantum training exhibits a better performance and generalization, with the accuracy in both the training and the test sets quickly approaching 100%, while the classical training tends to get stuck in local minima that lead to accuracies of approximately 80% in the training set, with lower ones in the test set, indicating poor generalization.

### 4.4 Comparative estimates of scaling

The different approaches to encoding the loss function presented here incur different computational costs in calculating the target Hamiltonian Ĥ and other gate complexities in the quantum circuit that implements the adiabatic time evolution.

It is worth comparing the different approaches to see how they scale with meta-parameters, e.g.., the number of hidden layers and the total number of qubits. To compare, we assume that Ĥ is decomposed as a polynomial in Pauli matrices to encode in a time-evolution circuit. The number of gates in the circuit will then be bounded from above by a quantity proportional to the number of terms *T* in this polynomial, multiplied by its degree *D*.

A Hamiltonian for a system of *N*_*q*_ qubits is in general an $2^{N_{q}}\times 2^{N_{q}}$ matrix. Thus, for the generic “matrix approach”, one needs to compute $2^{2N_{q}}$ quantities in the preparation stage of the calculation. The decomposition of Ĥ in terms of Pauli matrices will thus require $2^{2N_{q}}$ matrix multiplication and trace operations. Finally, the resulting polynomial in Pauli matrices will have degree $D=2^{2N_{q}}$ and roughly $T=2^{2N_{q}}$ terms, so the number of gates scales roughly as $2^{4N_{q}}$. However, the loss functions of neural networks typically lead to a very sparse Ĥ, so there is scope for significant improvement on these scalings.

Using the “Pauli-spin method” is one possible strategy to take advantage of sparsity. The maximum number of terms in Ĥ is then several chains of length $2^{N_{q}}$ of identity or Pauli *Z* matrices. One needs to compute the coefficient to each of these chains in Ĥ, thus the maximum number of quantities to compute in this approach is a factor $2^{N_{q}}$ that is smaller than in the general case. In practice, this number might be much smaller. Moreover, these quantities are computed directly by replacing the binary encoding of the weights with the loss function, without the need for decomposition of Ĥ into a basis involving matrix multiplications and traces. The degree of the Ĥ polynomial is, in this case, independent of the number of qubits and increases with the number of layers of the network, but not with the number of weights per layer.

Thus, the scaling of both *T* and *D* improves significantly for relatively shallow networks in the Pauli-spin approach. To simplify the discussion, we consider a neural network with *L* layers, all having a polynomial activation with degree *d*, and an *M* × *M* matrix of weights with no biases. The number of terms in the Ĥ polynomial is then


$$\begin{array}{l} T≲M^{d^{L}}\;. \end{array}$$

This can be shown by induction on *L*. For a network with a single layer, *L* = 1, the number of terms is bounded by the number of terms in a degree-*d* polynomial in *M* variables:


$$\begin{array}{l} T<\left(\begin{array}{c} d+M\\ d \end{array}\right)\overset{M\to \infty }{\sim }M^{d}\;. \end{array}$$

Similarly, adding a layer to an (*L* − 1)-layer network gives several terms bounded by the number of terms in a degree-*d* polynomial in variables that are degree-*M*^*d*^*L*−1^^ polynomials themselves. This equation shows that the number of terms, and thus the gate complexity, is polynomial in *M* in this approach. On the other hand, the scaling with the number of layers is much worse: it is doubly exponential. It will thus quickly saturate the generic bound for Pauli-spin encoded functions of $2^{N_{q}}$, so the latter is the stronger one for deep neural networks.

In the case of binary neural networks with step-function activations, the degree of the activation polynomials is *d* = *M*. Thus, the advantages over classical algorithms provided by their quantum training are obtained at the price of an *M*^*M*^ scaling of the number of terms with the number of weights per layer. A potential source for improvement on this front is binary activations with a lower degree or a lower number of terms. An example of such an activation would be one that required all inputs of the layer to be 1 for it to be 1, otherwise being 0.

In terms of real-world applications, there are already simple problems to which this training method would be applicable. One simple example in the realm of High Energy Physics is event classification for events generated by simulations of proton-proton collisions at the LHC. Abel et al. (2022b) for example, considered events in which the final state contains two top quarks. The classification was to determine whether (*y* = 1) or not (*y* = −1), with the signal corresponds to the two tops being the decay products of a hypothetical new particle, the so-called *Z*′, and with the background (corresponding to the label *y* = −1) corresponding to the signal being from Standard Model physics, and with the features being the highest transverse momentum of a *b*-jet and missing energy. Our method would clearly be applicable to this example as it has the same dimensionality of features. Extending to more interesting cases is a matter of scaling the network in the manner described above. For *N*_*f*_ features, we require at least $N_{q}\approx N_{f}^{2}$ qubits. Thus, the current bottle-neck is the *simulation* of such a large number of qubits which would restrict such an analysis to order five features or less, with the caveat being that the number of gates grows rapidly as $2^{4N_{q}}$. However, going beyond simulation on genuine devices would solve the former problem, with the main practical hurdle being fault tolerance across many gates. Therefore, due to the reduction in gate number discussed above, the Pauli-spin method appears to offer significant advantage for long-term prospects.

## 5 Conclusion

Neural networks are ubiquitous optimization tools used in science and everyday tasks. The most time and resource-consuming part of their design is the training process. In this study, we have demonstrated the potential of Adiabatic Quantum Computing as a powerful tool for training neural networks. Our study addresses the computational challenges encountered when classically training NNs. We have demonstrated that AQC can effectively be implemented on gate quantum computers to train neural networks with continuous and discrete weights, as well as the so-called binary networks. Our findings indicate that AQC offers a robust and efficient approach to finding the global minimum of the loss function, thereby optimizing the NN. It is then possible to extract the optimally trained network parameters for deployment as a classical neural network.

The proposed methodology involving the “matrix method” and the “Pauli-spin method” effectively encodes and solves this optimization problem. As we leveraged the Qibo package to facilitate fast and accurate quantum circuit evaluation, our approach is scalable and practical for near-term quantum devices.

Compared to previous quantum approaches, which were based on quantum annealing using a transverse Ising model Hamiltonian, the AQC approach that we have proposed in this article enhances the expressivity of the trained neural networks and expands the applicability of quantum training methods to the gate quantum computing paradigm. The limitation of this computational method for any optimization problem is purely technical, i.e., posed by the number of qubits and the coherence time of the quantum device. The method is, however, absolutely general and can be applied to any optimization task. Thus, we have provided scaling arguments to assess how severe the technical limitations are and how much future devices have to improve to be able to address real-life problems. Current devices and simulators, unfortunately, can only perform calculations for small-scale systems, but this is, in general, the limitation for any form of quantum computing proposed.

Extending this methodology to more complex neural network architectures and loss functions would be of interest in expanding its applicability to broader classes of problems. Thus, this approach opens up new avenues for harnessing the computational prowess of quantum computation in the realm of machine learning, particularly in the training of neural networks.

## Data availability statement

The original contributions presented in the study are included in the article/supplementary material, further inquiries can be directed to the corresponding author.

## Author contributions

SA: Conceptualization, Investigation, Methodology, Software, Writing – original draft, Writing – review & editing. JC: Conceptualization, Investigation, Methodology, Software, Writing – original draft, Writing – review & editing. MS: Conceptualization, Investigation, Methodology, Software, Writing – original draft, Writing – review & editing.
